# Mood and Global Symptom Changes among Psychotherapy Clients with Depressive Personality

**DOI:** 10.1155/2012/208435

**Published:** 2012-12-12

**Authors:** Rachel E. Maddux, Lars-Gunnar Lundh

**Affiliations:** Department of Psychology, Lund University, P.O. Box 213, 221 00 Lund, Sweden

## Abstract

The present study assessed the rate of depressive personality (DP), as measured by the self-report instrument depressive personality disorder inventory (DPDI), among 159 clients entering psychotherapy at an outpatient university clinic. The presenting clinical profile was evaluated for those with and without DP, including levels of depressed mood, other psychological symptoms, and global severity of psychopathology. Clients were followed naturalistically over the course of therapy, up to 40 weeks, and reassessed on these variables again after treatment. Results indicated that 44 percent of the sample qualified for DP prior to treatment, and these individuals had a comparatively more severe and complex presenting disposition than those without DP. Mixed-model repeated-measures analysis of variance was used to examine between-groups changes on mood and global severity over time, with those with DP demonstrating larger reductions on both outcome variables, although still showing more symptoms after treatment, than those without DP. Only eleven percent of the sample continued to endorse DP following treatment. These findings suggest that in routine clinical situations, psychotherapy may benefit individuals with DP.

## 1. Introduction

Understanding the reasons why individuals respond or do not respond to treatments for psychological problems is a topic of interest to clinicians and researchers alike. Research examining psychotherapy outcomes [1–3], psychopharmacology outcomes [4], as well as protocols that include combination treatment arms [5–7] have consistently shown that while many patients and clients improve over the course of treatment, many remain unwell [8–10]. 

 In recent years, focus has turned toward personality as one possible predictor of outcome [11]. This is because many individuals with primary clinical disorders simultaneously present with Axis-II conditions [12–15]. Conventional clinical wisdom often holds that those with entrenched, maladaptive personality traits represent an extra challenge in the treatment setting, perhaps because of difficulties in the working alliance [16, 17] or issues involving treatment compliance [18–20]. Moreover, compared to clients without personality disorders (PD), clients with PDs have more severe psychopathology [21, 22], generally take longer to treat [23–25], and are more difficult to treat [26, 27]. 

 One PD that has received attention is Depressive Personality Disorder (DPD). DPD, according to the *Diagnostic and Statistical Manual of Mental Disorders, Fourth Edition* (DSM-IV; [28]), is characterized by a pervasive pattern of depressive cognitions and behaviors beginning by early adulthood and present in a variety of contexts, as indicated by at least 5 of the following 7 criteria: (1) usual mood is dominated by dejection, gloominess, cheerlessness, joylessness, unhappiness; (2) self-concept centers around beliefs of inadequacy, worthlessness, and low self-esteem; (3) is critical, blaming, and derogatory toward self; (4) is brooding and given to worry; (5) is negativistic, critical, and judgmental toward others; (6) is pessimistic; (7) is prone to feeling guilty or remorseful. The diagnostic criteria also stipulate that this pattern should “not occur exclusively during Major Depressive Episodes and is not better accounted for by Dysthymic Disorder” [28].

 Although many have debated the discriminability of DPD from the chronic, mild depressive condition Dysthymic Disorder (DYS; [29, 30]) and Major Depressive Disorder [31], research has shown that DPD and depression are two overlapping yet distinct clinical entities [32, 33]. The presence of DPD has been identified in various studies however as a precipitating risk marker for the development of depression [34–37], and it appears to impact its course—predicting less change in depressive symptoms [38] and contributing to a lower likelihood of episode remission [39]. Further, DPD is commonly observed in clinical research settings [39–42] and a depressive-dysphoric personality type has been recognized by practicing clinicians as the largest patient group—about 20%—of those with personality pathology [43]. Thus, identifying individuals presenting for treatment with DPD may have important prognostic value for case conceptualization and treatment planning.

With respect to treatment, only a handful of studies have examined the influence of DPD on outcomes, and these studies have focused solely on depression [24, 44, 45]. In a secondary analysis of a large, multi-site clinical trial, Maddux and colleagues [46] examined the moderating effect of DPD, as diagnosed by the clinician-rated Structured Clinical Interview for Axis-II Disorders (SCID-II; [47]), on levels of depression following 12 weeks of treatment with an antidepressant medication (ADM), a modified version of cognitive-behavioral psychotherapy, or their combination among 680 patients with chronic forms of depression. Results indicated no significant differences between those with and without DPD in terms of their response to any modality of treatment. DPD itself however was not reassessed at endpoint, so it was not possible to gauge whether treatment affected DPD specifically. 

More recently, Ryder et al. [48] reported results from a trial that examined DPD, as measured by the SCID-II self-report [49], as a predictor of overall and preferential treatment outcome for 120 patients with major depression. Patients in this study were randomized to 16–20 weeks of treatment with cognitive-behavioral therapy (CBT), interpersonal therapy (IPT), or ADM which included 7 possible medications (bupropion, citalopram, fluoxetine, paroxetine, phenelzine, venlafaxine, or sertraline) in flexible dose ranges. Results indicated that DPD did not predict overall treatment outcome; however, a pattern of preferential responding did emerge. DPD was associated with a worse outcome to IPT, but not to CBT or ADM. Further, individuals identified as high on DPD traits (≥5 DPD symptoms) treated with IPT had a significantly poorer remission rate (27%) as compared to those identified as low on DPD traits (<5 DPD symptoms; 77%). As the authors of this study note, this is “a dramatic illustration of the potential clinical utility of information about DP traits” (pp. 400).

The present study aims to examine depressive personality (DP) in *a naturalistic* treatment setting, wherein clients scheduled to begin psychotherapy sessions at an outpatient clinic are assessed prior to and at the termination of treatment. Because we employed a self-assessment measure of DPD, and a diagnosis of DPD should not be made only on the basis of self-rating, we will refer to the construct studied as “depressive personality” (DP) rather than DPD. More specifically, the purpose was to (a) study how common DP was in this clinical sample, (b) evaluate differences in demographic and clinical characteristics, including the presenting symptom profile, between those with and without DP, (c) determine the relationship between DP and depressed mood and global symptom severity (GSI), (d) test whether DP groups differ in terms of their changes from pre- to posttreatment on levels of depressed mood and GSI within two modalities of psychotherapy (i.e., using DP as a *categorical* variable) and (e) gauge whether DP may predict treatment outcomes (i.e., using DP *dimensional *scores).

## 2. Method

### 2.1. Setting

The present study took place within the psychotherapy clinic at the Department of Psychology, Lund University, Sweden. At this clinic, students who are in their 4th and 5th years of the 5-year psychologist training program treat patients while being supervised by experienced, licensed psychologists. The Department of Psychology at Lund University is one of the largest departments at the Faculty of Social Sciences with about 110 full-time staff members and about 1,200 students at different levels of training. The 5-year (10 semesters) psychologist training program accepts 86 new students per year and the competition is very strong, with an admittance rate of only 5%.

The psychotherapy course is spread over six semesters of the psychologist training program, starting on the fifth semester and ending at the tenth semester. During the seventh semester, half of the students start by practicing cognitive-behavioral therapy (CBT) for one year, and then shift to psychodynamic therapy (PDT) during the next year, whereas the other half of the students start with PDT and then shift to CBT during their last year. Although University affiliated, the clinic primarily serves members of the community who present with clinical disorders typical of an outpatient clinic. There are minimal exclusion criteria for services; however, individuals with psychotic disorders or who are otherwise deemed unstable by intake psychologists (licensed staff, not trainees) are referred out to alternative mental health settings.

### 2.2. Participants

From August 2008 to February 2009, all new clients who completed an intake interview with a staff psychologist and were scheduled to begin sessions of psychotherapy at the clinic were informed about the study. Of these 180 individuals, 160 agreed to participate (89% response rate). Participation included completing a self-report questionnaire packet following the intake interview (or at home with a mail-in option). This packet contained a demographics form, childhood and adult history of psychological problem and treatment, family history of psychological problem and treatment, the *Symptom Checklist-90* (SCL-90; [50]), and the *Depressive Personality Disorder Inventory *(DPDI; [51]). The therapists were blind to the client's scores on these instruments. Apart from the background and history forms, clients were also asked to complete these questionnaires after their termination session.

DPDI data were unavailable for one client, resulting in a final sample of 159 at pretest. Of these, 61% (*n* = 97) would undergo CBT and 39% (*n* = 62) would undergo PDT. During the intake interview, clients were informed about the two kinds of therapy that were available (cognitive-behavioral therapy and psychodynamic therapy) and were guided to make a choice between these; if a client stated no preferences, the staff psychologist referred him or her to the kind of therapy that was considered most suitable. The number of psychotherapy sessions for each client depended on his/her needs (although no therapy could last longer than 40 weeks due to therapists' training schedules). The mean number of months spent in psychotherapy for the full sample was 6.5 (SD = 2.92), with those in CBT spending 6.1 months (SD = 2.54) and those in PDT spending 6.9 months (SD = 3.30) on average. Full posttreatment data were available for 97 clients with both SCL-90 depression subscale scores and SCL-90 GSI scores. All statistical analyses were carried out on this completer sample.

### 2.3. Measures

*The Depressive Personality Disorder Inventory *(*DPDI;* [51]). The DPDI is a 41-item self-assessment inventory with a 7-point response format (1 = “totally agree” to 7 = “totally disagree”). Higher scores reflect a stronger endorsement of depressive personality, and a total scale score of 170 has been suggested as the categorical cut-off for a positive indication of DP. Using this convention, Huprich et al. [52] found that individuals could be accurately identified with strong diagnostic efficiency (sensitivity = .82; specificity = .80; positive predictive power = .75; negative predictive power = .86; overall diagnostic power = .81). 

The DPDI has shown good reliability in a number of studies, with Cronbach's alpha values ranging from .91–.94 in nonclinical samples [51, 52] to .95-.96 in samples of psychiatric outpatients and community mental health respondents [52, 53]. It has also evidenced good convergent validity, correlating with both a semistructured interview (Diagnostic Interview for Depressive Personality (DIDP) [54]; *r* = .72 undergraduates, .61 psychiatric outpatients [52]; *r* = .51 [53]) and an alternate self-report (SCID-II-SR; [49]; *r* = .75 [52]; *r* = .72 [53]). Two- and five-week test-retest reliabilities for the DPDI have recently been reported at .89 and .82, respectively [55]. The DPDI was translated into Swedish by Maddux et al. [56], who showed the Swedish version to have good reliability and validity.

In this study, the same categorical cut-off conventions suggested by Huprich et al. [52] were used. A total scale score of 170 or higher was considered indicative of DP. Because the DPDI is a self-report and not clinician-rated diagnostic instrument, we refer to this as merely as a cut-off for DP and not a formal diagnosis of DPD. Internal consistency reliability for the DPDI in this sample was *α* = .94. 

*Symptom Checklist-90 *(*SCL-90; *[50, 57]). The SCL-90 is a widely used self-report symptom inventory, originally developed to evaluate psychological symptom patterns among psychiatric and medical patients. It contains 90 items, which ask how much the respondent has suffered from various symptoms during the past week, each rated on a five-point scale of severity from 0 (none) to 4 (extreme). These items measure nine primary symptom dimensions of psychological distress, forming the following subscales: (1) Somatization; (2) Obsessive-Compulsive; (3) Interpersonal Sensitivity; (4) depression; (5) Anxiety; (6) Hostility; (7) Phobic Anxiety; (8) Paranoid Ideation; (9) Psychoticism. Each dimension comprises 6–13 items, which are averaged to provide a mean rating for the dimension. Three global indices are also included; the global severity index (GSI; overall level of psychological distress as indicated by the mean of all 90 items), the positive symptom distress index (PSDI; intensity of symptoms endorsed as indicated by the mean of all items scored above zero), and the positive symptom total (PST; count of items scored above zero). Internal consistency coefficients of the SCL-90 subscales and global indices across different populations have ranged from *α* = .77–.90 [58]. The test-retest reliability has also been established, ranging from .78–.90 among psychiatric outpatients over one week [59] and .68–.80 over a 10-week interval [60]. 

The SCL-90 has been employed to measure psychological symptom distress and change over time in numerous psychopharmacological trials [58, 61–64] and psychotherapy treatment studies [65–71]. In this study, Cronbach's alpha values were *α* = .85 (somatization), *α* = .85 (obsessive-compulsive), *α* = .84 (interpersonal sensitivity), *α* = .90 (depression), *α* = .84 (anxiety), *α* = .80 (hostility), *α* = .75 (phobic anxiety), *α* = .77 (paranoid ideation), *α* = .78 (psychoticism), and *α* = .97 (GSI). The depression subscale and GSI served as the primary outcome variables.

### 2.4. Statistical Analyses

Statistical procedures were conducted using SPSS statistical package for Windows, Version 18.0. Differences in basic demographics and clinical characteristics between-groups (DP and no DP) were tested using independent samples *t*-test or chi-square analyses. Relationships between DPDI scores and SCL-90 depression and SCL-90 GSI scores at pre- and posttreatment were evaluated via bivariate correlation. Analyses of change between-groups from pre- to posttreatment on levels of depression and GSI were conducted using mixed-model repeated-measures analysis of variance (MMRM ANOVA). Between-subjects factors were DP Group (DPyes or DPno) and Treatment Type (CBT or PDT). The within-subjects factor was Time (pre- and postscores). To evaluate whether DP could predict outcomes above and beyond pretreatment depression or GSI levels, hierarchical regressions were performed: SCL-90 pretreatment depression scores were entered in step 1 and DPDI pretreatment scores in step 2, with SCL-90 posttreatment depression scores serving as the criterion variable. This was repeated using SCL-90 GSI scores at pre- (step 1) and posttreatment (criterion variable).

## 3. Results

### 3.1. Demographics and Clinical Characteristics

Of the full sample (*N* = 159), 75% (*n* = 119) were women. Ages ranged from 19 to 63 years old, with an average age of 30 (SD = 9.02). Forty six percent (*n* = 73) indicated their relationship status as alone without a partnership, 38% (*n* = 61) married, and 16% (*n* = 25) alone but in a relationship. Fifty-eight percent (*n* = 92) reported being a member of the community utilizing the Univeristy clinic, while the remaining participants were active students. The overall mean score on the DPDI was 160.53 (SD = 37.85) prior to treatment. Those in the CBT group scored slightly higher (*n* = 97; *M* = 164.49, SD = 35.89), although not significantly so, than those in the PDT group (*n* = 62; *M* = 154.32, SD = 40.24). Using the categorical cut-off score of 170 on the DPDI, 44% (*n* = 70) of the sample qualified for DP at pretreatment (*n* = 47 in CBT, *n* = 23 in PDT). Those with DP spent approximately one month longer in therapy (*M* = 9.11, SD = 2.79) as compared to those without DP (*M* = 7.96, SD = 3.22), though this was not a statistically significant group difference. In PDT treatment, those with DP spent longer (*M* = 9.00, SD = 2.75) but not significantly so compared to those without DP (*M* = 8.85, SD = 3.18); while in CBT treatment, those with DP spent significantly longer in therapy (*M* = 9.17, SD = 2.87) than their non-DP counterparts (*M* = 7.11, SD = 3.07; *t*(49) = −2.46, *P* < .05). Comparisons between those with and without DP on demographic and clinical variables can be found in Table 1. A Bonferroni correction was applied to the multiple comparisons of the SCL-90 scales.

### 3.2. Relationship between Depressive Personality and Psychological Symptoms

Table 2 presents the correlations between measures of DP, depressed mood, and GSI at pre- and posttreatment. As seen in the table, DP showed high correlations with depressed mood at both pretest and posttest (*r*s .72–.81).

### 3.3. DP and Symptom Changes

Two variables were of primary interest with respect to treatment outcomes: depressed mood and GSI levels. Those with DP displayed higher pretreatment (*M* = 2.56, SD = 0.69) and posttreatment levels of depressed mood (*M* = 1.57, SD = 0.91) as compared to those without DP (*Mpre* = 1.30, SD = 0.70; *Mpost* = 0.93, SD = 0.61). Likewise, those with DP displayed higher pretreatment (*M* = 1.67, SD = 0.54) and posttreatment GSI levels (*M* = 1.01, SD = 0.52) as compared to those without DP (*Mpre* = 0.88, SD = 0.47; *Mpost* = 0.64, SD = 0.39). When naturally occurring groups (i.e., non-randomized) exhibit such differences prior to treatment, it likely reflects some meaningful, substantive differences that are attributable to group membership; therefore, attempts to mathematically modify (control/covary out) the variable are considered inappropriate [72, 73]. Thus, mixed-model repeated-measures analysis of variance (MMRM ANOVA) was selected as the data analytic method. Assumptions for MMRM were checked to ensure no violations occurred. Levene's Test of equality of error variances was significant for posttreatment depressed mood (*F*(3, 94) = 7.903, *P* = .00), however because the largest variance was no more than four times the smallest, the analysis is most likely valid [74]. In this case, the largest variance was approximately twice the smallest indicating the violation was not severe. 

**Figure 1:**
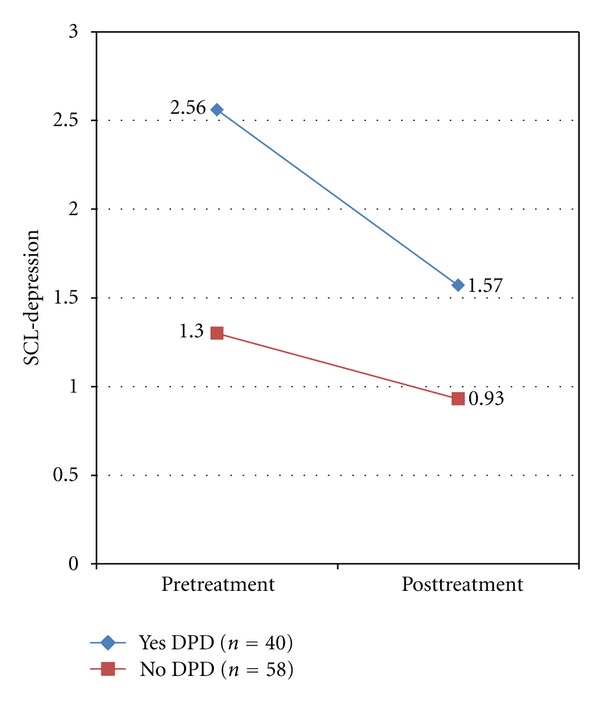
Changes in mean depression scores from pre- to posttreatment between groups (DP yes, top line; DP no, bottom line). Note. SCL-Depression: symptom checklist-90 depression subscale.

MMRM ANOVA was employed to determine whether those with and without DP would show differential changes in depressed mood (analysis 1) and/or GSI (analysis 2) from pre- to posttreatment, and whether any difference would depend on type of treatment received. In analysis 1, DP Group (yes or no) and Treatment Type (CBT or PDT) were entered as between-subjects factors, and Time (pre- to postdepression scores) was entered as the within-subjects factor. Results of this analysis found a significant interaction between DP group and time (*F*(1,94) = 11.22, *P* < .01, partial *η* ^2^ = .12), indicating those with DP experienced a statistically greater reduction in depressed mood as compared to those without DP (see Figure 1). Tests of simple effects showed groups had significantly different levels of depressed mood at both pretreatment (*F*(1,96) = 78.39, *P* < .01) and posttreatment (*F*(1,96) = 17.65, *P* < .01). As reported previously, those with DP showed comparatively higher scores on depressed mood at both time points. Tests of simple effects also indicated both groups changed significantly over time (DP [*F*(1,96) = 55.73, *P* < .01]; noDP [*F*(1,96) = 11.33, *P* < .01]). There was no significant interaction between treatment type and time (*F*(1,94) = 0.86, *P* = .35) nor a significant 3-way interaction between DP group × treatment type × time (*F*(1,94) = 0.93, *P* = .34), suggesting the differential changes in depressed mood found over time between DP groups are not dependent on the treatment modality.

**Figure 2:**
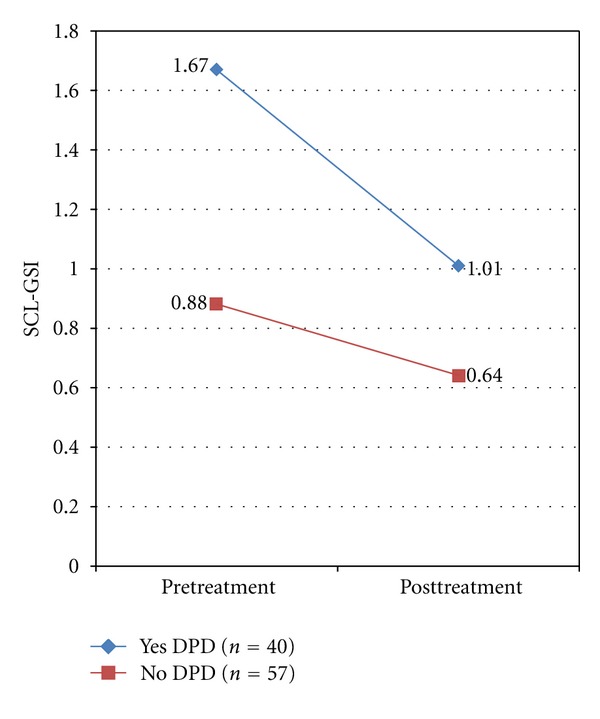
Changes in mean GSI scores from pre- to posttreatment between groups (DP yes, top line; DP no, bottom line). Note. SCL-GSI: Symptom Checklist-90 Global Severity Index.

In Analysis 2, DP group (yes or no) and treatment type (CBT or PDT) were entered as between-subjects factors, and time (pre- and post-GSI scores) was entered as the within-subjects factor. Results of this analysis found a significant interaction between DP group and time (*F*(1,93) = 15.34, *P* < .01, partial *η* ^2^ = .14), indicating those with DP experienced a statistically greater reduction in GSI scores as compared to those without DP (see Figure 2). Tests of simple effects showed groups had significantly different GSI levels at both pretreatment (*F*(1,95) = 58.73, *P* < .01) and posttreatment (*F*(1,95) = 16.37, *P* < .01). As reported previously, those with DP showed comparatively higher GSI scores at both time points. Tests of simple effects also indicated both groups changed significantly over time (DP [*F*(1,95) = 70.89, *P* < .01]; noDP [*F*(1,95) = 13.10, *P* < .01]). There was no significant interaction between Treatment Type and Time (*F*(1,93) = 1.89, *P* = .17) nor a significant 3-way interaction between DP group × treatment type × time (*F*(1,93) = 0.111, *P* = .74). These results suggest that the differential changes in GSI levels found over time between DP groups are not dependent on the treatment modality. Only eleven percent of the sample (*n* = 18) continued to qualify for DP following treatment.

### 3.4. DP as a Predictor of Outcome

To determine whether DP may serve as a unique predictor of posttreatment depressed mood and/or GSI, two hierarchical regressions were performed. In regression 1, pretreatment scores on depressed mood were entered in the first step, and pretreatment DPDI scores in the second step, with posttreatment scores on depressed mood as the outcome variable. Results (see Table 3) show that DPDI scores accounted for a small but significant portion of the variance in posttreatment scores on depressed mood; that is, higher pretreatment DPDI scores predicted higher levels of depressed mood after treatment, *independently of clients' pretreatment level of depressed mood*. In regression 2, pretreatment GSI scores were entered in the first step, and pretreatment DPDI scores in the second step, with posttreatment GSI scores as the outcome variable. Results (Table 3) show that DPDI scores did not account for a significant portion of the variance in posttreatment GSI scores.

## 4. Discussion

Clients with a depressive/dysphoric type of personality are frequently seen in clinical practice, representing approximately 20% of all patients according to experienced, practicing psychiatrists, and psychologists [43]. Investigating DP in clinical samples therefore seems prudent, and determining whether these individuals differ in clinical presentation and response to treatment as compared to those without DP is clinically relevant information for case conceptualization and treatment planning.

In this study, a large portion of clients (44%) beginning psychotherapy treatment for various psychological problems scored at or above 170 on the DPDI, suggesting the presence of DP. This is similar to the rate found in other studies of clinical samples using the DPDI [52, 53]. The majority of those with DP presented at intake citing depression as the primary presenting problem (61%), which was nearly twice the rate of those without DP (32%). From these data, it is not possible to decipher whether DP put these individuals at higher risk for depression; however, it is clear that cross-sectional comorbidity between DP and depressed mood is substantial. Conversely, these data also demonstrate that DP and depressed mood can exist to some degree independently, a controversial topic [75], as not all depressed individuals simultaneously qualified for DP. 

These data reflect a current discussion in the literature about the general relationship between PDs and depression [76, 77], and point to the many challenges facing mental health leaders about how best to conceptualize, assess, and diagnose personality pathology in the next version of the DSM [78]. A large conceptual reformulation is currently underway for personality and personality disorder categories for DSM-5 [79]. Different models have been proposed, including prototypes and dimensional trait ratings, and it is possible that the current DPD categorical diagnosis will be reconfigured in some new way. Until formal decisions are made, it is not possible to know how DPD will be represented, if at all; however, clinicians [43] and expert researchers [80] both acknowledge the high rates of DPD seen in clinical settings, and this research substantiates that data. 

Regarding other aspects of clinical presentation, compared to clients without DP, those with DP had more severe psychopathology across all subscales and indices measured prior to treatment, including levels of depression, anxiety, somatization, obsession-compulsion, interpersonal sensitivity, hostility, phobic anxiety, paranoid ideation, and psychoticism as well as global levels of symptom severity, positive number of symptoms, and overall psychological distress. Thus for those presenting with DP, they also appear to experience a host of other distressing pathology prior to treatment. 

Results from the mixed-model ANOVA revealed that despite higher scores on depressed mood and GSI at pre- and posttreatment, those with DP made comparatively greater gains over the course of time regardless of treatment modality. This means that, although in one way clients had poorer outcomes (i.e., higher end of treatment scores) in another way those with DP did better (i.e., showing more dramatic reduction in symptoms). This piece of data is quite crucial, such that clients with DP can be shown not only to improve over the course of psychotherapy, but their rate of improvement is greater than those without DP. 

This finding seems somewhat counterintuitive particularly because of the long-standing presumption that personality pathology represents an obstruction to successful treatment outcomes, and indeed some research studies have supported this contention [81–83]. However, a growing body of evidence now points in the opposite direction, indicating instead that comorbid PDs do not stymie treatment efficacy [84–86]. In an applied clinical context similar to the study presented in this paper, Saulsman and colleagues [45] found that compared to those classified as low DP, those with high DP also had higher pre- and post-endpoint depression scores, although the rate of improvement over a 10-week group CBT mood management intervention was found to be similar between groups. The authors concluded from this research that DP does not negatively affect psychotherapy for depression (despite higher endpoint scores), results that are in line with the findings of the present study. 

There is an important caveat to note however, which is that in the present study the time spent in therapy was approximately one month longer for those with DP than without DP, and nearly 2 months longer for those with DP who were receiving CBT. (Those with and without DP spent roughly the same number of months in PDT). So while overall outcomes were undifferentiated by treatment modality, the length of therapy was a bit longer for those with DP receiving CBT than those without DP receiving CBT. This means that CBT may be an effective approach for clients with DP, but may require some extra sessions to achieve equivalent results. 

One important question generated from this study is whether or not DP is a concomitant of a depressed mood (i.e., a mood state effect) that presents prior to treatment and simply resolves in parallel with the amelioration of the mood episode. As was evidenced by the correlational analysis, strong positive associations were found between DP and depressed mood at pre- and posttreatment. In order to help disentangle this, we conducted a set of regression analyses examining the extent to which DP pretreatment scores may predict depressed mood and GSI scores at posttreatment, *above and beyond* that which is predicted by pretreatment levels of depressed mood and GSI, respectively. Here we found that DP could, in fact, predict depressed mood outcomes independently, although it could not predict GSI outcomes independently. This lends some credence to the notion that DP indeed exists as a mood-independent construct, and it should possibly be conceptualized more as a trait marker that is itself affected by treatment strategies.

Also, as summarized by Huprich [87], previous research clearly shows that mood state affects what patients recall and report about themselves, which poses a challenge to the assessment of DPD. Although the high correlations between depressed mood and DPDI scores in the present study are quite consistent with the assumption that depressed mood *affects* how one responds to instruments like the DPDI, it is important to note that the results also showed that DPDI predicted posttest depressed mood even when controlling for pretest depressed mood. This clearly indicates that DPDI does measure something more than *merely* depressed mood.

Interestingly, related research examining changes in neuroticism and extraversion—two higher order trait components conceptually related to DP [88–91]—provided evidence that pharmacological treatment and to a lesser extent psychological treatment appears to have a specific effect on personality that is distinct from its effect on depression [92]. As the authors of this study conclude, replication of these results could disconfirm the state-effect hypothesis and instead support the notion that the effects of treatment on personality go beyond and perhaps contribute to their antidepressant effects. Extant psychopharmacology research has supported this notion as well [14, 93, 94], as has research examining the therapeutic benefits of both psychodynamic and cognitive-behavioral treatment on personality disorders [95]. In fact, there is some data to suggest personality pathology mediates changes in depression severity in response to treatment, such that any treatment effect on depression occurs via personality pathology reduction [96]. Future research on the pathways and mechanisms of change await. 

There are important limitations to the present study that warrant discussion. First, the study was conducted in a naturalistic environment which has the advantage of a real-world clinical situation; however, such a design therefore lacked true experimental conditions. Clients, for example, were not randomized into treatment groups. Further, it was not possible to draw conclusions about whether treatment itself was definitively causal in the changes in depressed mood, GSI, or DP which occurred from pre- to posttreatment. Future studies aiming to maximize internal validity could employ a randomization procedure for treatment conditions and a control group, and/or employ a design that matches those with and without DP to the various conditions. This study has its primary strength in external validity however, as the results can be generalized to other similar outpatient clinical settings. 

A second limitation to be acknowledged is that psychotherapy beyond the intake session was conducted by graduate level trainees. These clinicians are novice, nonlicensed practitioners in training; however, they are in the final stages of their program and work directly under the supervision of staff psychologists. Results from this study demonstrate that supervised trainees appear to deliver treatment in a successful manner, which are outcomes that have been similarly obtained in several other research studies [97–99].

A third limitation is that posttreatment data was not available for all clients who participated in the research. This is primarily due to the naturalistic design of the study coupled with graduate trainees schedules. Trainees were asked to alert the research staff when his/her client became scheduled for a termination session, at which time follow-up data would be collected. In some cases, trainees forgot, clients forgot, or the research team had logistic difficulty garnering follow-up assessments. As a strategy to bolster postdata collection, reminder letters were sent to all clients who terminated but did not complete posttreatment assessment; nonetheless, only 97 clients completed the full assessment. Future research operating in naturalistic settings (i.e., not with a priori defined treatment endpoints) could benefit from termination session research protocols or procedures, to be certain data is collected when individual clients conclude therapy at different times. 

 A final limitation to this research is that the assessment of DP occurred solely by way of a self-report questionnaire. The DPDI however has been employed in numerous nonclinical and clinical studies [87], has been cross-culturally, psychometrically replicated [56], and research supports its reliability, convergent, and construct validity [51–53, 55]. However, there remain some problems with the measure in terms of its discriminant validity [100], and as the developer of the instrument has recommended (see [80]), it may be necessary to expand the range of assessment tools (e.g., performance-based measures) when DPD presents clinically alongside depressive symptoms. Thus, future work could confirm DP through clinical interviewing, or perhaps with a second assessment using a different measure. Further, when in a state of psychological distress, individuals can describe themselves as having more severe personality pathology than when in a premorbid or intermorbid state [101]. Thus, taken together, it seems prudent to employ pluralistic measurement procedures in order to be certain DP is present and a diagnosis is warranted.

## 5. Conclusions

The purpose of this research was to investigate the rate of depressive personality among clients entering treatment at a university-based psychotherapy clinic. Further, we aimed to determine whether those with DP presented for treatment with a differential symptom profile than those without DP as well as to garner information about their comparative treatment outcomes. Results indicated that nearly half of clients (44%) entering treatment qualified for DP, and compared to those without, those with DP had a more severe and complex presenting disposition across a range of psychopathology. Those with DP showed greater depressed mood and overall psychopathology both at treatment start and termination. However, they also made greater gains on these variables over the course of time, and by treatment end only 11% of clients endorsed DP. These results together suggest that psychotherapy may be beneficial for individuals who have DP as a part of their clinical presentation. 

## Figures and Tables

**Table 1:** Demographics and clinical characteristics for full sample (*N* = 159) split by DP.

	Yes DP	No DP	*χ* ^2^ or *t*
	(*n* = 70, 44%)	(*n* = 89, 56%)	
	*n* (%)	*n* (%)	
Gender			
Female	55 (79%)	64 (72%)	*χ* ^2^ = ns
Male	15 (21%)	25 (28%)	
Age			
Range	19–54	20–63	*t* = ns
Mean	28.36 (±7.75)	30.65 (±9.83)	
Marital status			
Single, living alone	37 (53%)	36 (40%)	*χ* ^2^ = ns
Married	6 (9%)	20 (23%)	
Partner, living together	16 (23%)	19 (21%)	
Partner, living alone	11 (16%)	14 (16%)	
Referral			
Community member	38 (54%)	54 (61%)	*χ* ^2^ = ns
Active student	32 (46%)	35 (39%)	
Presenting problem			
Depression^a^	43 (61%)	28 (32%)	*χ* ^2^ (4) = 15.26*
Anxiety	8 (11%)	18 (20%)	
Relationship	4 (6%)	15 (17%)	
Other	8 (11%)	15 (17%)	
Comorbid^b^	7 (10%)	13 (15%)	
Concurrent tx			
Yes medication	19 (27%)	16 (18%)	*χ* ^2^ = ns
Yes med + other	3 (4%)	4 (5%)	
Yes other	2 (3%)	3 (3%)	
Previous adult tx			
Yes	45 (64%)	51 (57%)	*χ* ^2^ = ns
If yes, most recent adult problem			
Depression	27 (60%)	26 (51%)	*χ* ^2^ = ns
Anxiety	4 (9%)	11 (22%)	
Relationship	4 (9%)	7 (14%)	
Other	10 (22%)	7 (14%)	
Childhood treatment hx			
Yes	15 (21%)	11 (12%)	*χ* ^2^ = ns
Parent psyc history			
Yes	26 (37%)	44 (49%)	*χ* ^2^ = ns
Unsure	22 (31%)	24 (27%)	
DPDI pretest^ c^	194.96 (±17.50)	133.45 (±25.44)	*t*(154.44) = 18.02**
SCL-90			
Somatic	1.17 (±0.67)	0.79 (±0.75)	*t* (157) = 3.29**
OC	2.09 (±0.71)	1.25 (±0.76)	*t* (157) = 7.08**
Int Sen^c^	1.91 (±0.78)	0.97 (±0.63)	*t* (130.47) = 8.25**
Depression	2.49 (±0.71)	1.38 (±0.74)	*t* (157) = 9.65**
Anxiety	1.75 (±0.71)	1.17 (±0.72)	*t* (157) = 5.08**
Hostility^c^	1.08 (±0.79)	0.60 (±0.60)	*t* (125.83) = 4.18**
Phobic Anx	0.68 (±0.60)	0.36 (±0.57)	*t* (157) = 3.48**
Para Ideation^c^	1.24 (±0.81)	0.58 (±0.55)	*t* (115.62) = 5.78**
Psychoticism^c^	1.09 (±0.68)	0.49 (±0.47)	*t* (117.04) = 6.27**
GSI	1.59 (±0.51)	0.92 (±0.51)	*t* (157) = 8.25**
PSI^c^	57.96 (±12.29)	40.64 (±15.63)	*t* (155.98) = 7.80**
PDTI	2.42 (±0.41)	1.93 (±0.46)	*t* (157) = 6.99**

*P < .01, **P < .008 Bonferroni corrected.

^a^Standardized residuals > ±1.96 for depression as the presenting clinical problem.

^b^Comorbid problems, not including depression.

^c^Equal variances not assumed.

Note: DP: depressive personality; Univ: University; Psyc: psychological/psychiatric treatment; DPDI: depressive personality disorder inventory; OC: obsessive compulsive; Int Sen: interpersonal sensitivity; Anx: anxiety; Para: paranoid; GSI: global severity index; PSI: positive symptom index; PDTI: positive symptom distress index.

**Table 2:** Correlations between DPDI, Depression, and GSI at pre- and posttreatment.

Variable	1	2	3	4	5	6
1 DPDI (1)	—					
2 DPDI (2)	.688*	—				
3 SCL-D (1)	.721*	.466*	—			
4 SCL-D (2)	.526*	.812*	.485*	—		
5 SCL-GSI (1)	.674*	.400*	.858*	.409*	—	
6 SCL-GSI (2)	.544*	.769*	.519*	.894*	.585*	—

*P < .01.

Note. DPDI: depressive personality disorder inventory; SCL-90: symptom checklist 90; D: depression; GSI: global severity index; (1): administered at pretreatment; (2): administered at posttreatment.

**Table 3:** Multiple regression analyses: DPDI as a predictor of outcome.

Analysis 1: depression	*B *	SE *B *	**β**
Step 1			
** **Constant	.430	.158	
** **SCL-90 depression^a^	.418	.077	.483*
Step 2			
** **Constant	−.336	.319	
** **SCL-90 depression^a^	.160	.121	.184
** **DPDI^a^	.008	.003	.381*

Analysis 2: global symptoms	*B *	SE *B *	**β**

Step 1			
** **Constant	.265	.087	
** **SCL-90 GSI^a^	.439	.064	.577*
Step 2			
** **Constant	−.336	.319	
** **SCL-90 GSI^a^	.306	.095	.403*
** **DPDI^a^	.003	.002	.233

Analysis 1:** **R^2^ = .223 for Step  1, ΔR^2^ = 0.056 for Step  2 (*P* < .01).

Analysis 2: *R* ^2^ = .333 for Step  1, ΔR^2^ = 0.024 for Step  2 (*P* = .07).

*P < .01.

^a^Scores at pretreatment.

Note. DPDI: depressive personality disorder inventory; SCL: symptom checklist-90; GSI: global severity index.
